# Definition for AF course

**DOI:** 10.1002/clc.23409

**Published:** 2020-06-16

**Authors:** David E. Winchester

**Affiliations:** ^1^ Malcom Randall VAMC, Cardiology Section University of Florida College of Medicine, Division of Cardiovascular Medicine Gainesville Florida USA

To the editor,

In a recently published investigation, He et al demonstrated the risk associated with left atrial appendage (LAA) morphology and thrombosis.^1^ Specifically, they adopted a binary approach of “simple” and “complex” LAA morphology which may make it easier to apply in clinical practices compared to other proposed morphology classification systems. In the published logistic regression model, LAA morphology was associated with thrombosis risk; however, the conclusions of the manuscript fail to highlight that both nonparoxysmal atrial fibrillation and “AF course” have greater risk associations. “AF course” is of particular interest because the odds ratio was 55 for the lower end of the 95% confidence interval estimate; however, it is not defined in the manuscript. Readers and researchers interested in atrial fibrillation would benefit from knowing how “AF course” was defined.
